# COMMUNITY–CLINICAL LINKAGE MODELS FOR COMMUNITY-DWELLING OLDER ADULTS: A RAPID SCOPING REVIEW

**DOI:** 10.1093/geroni/igad104.2778

**Published:** 2023-12-21

**Authors:** Miriam Gofine, Gregory Laynor, Rachel Massar, Antoinette Schoenthaler

**Affiliations:** NYU Grossman School of Medicine, New York City, New York, United States; NYU Grossman School of Medicine, New York City, New York, United States; NYU Grossman School of Medicine, New York City, New York, United States; NYU Grossman School of Medicine, New York City, New York, United States

## Abstract

About 10% of older adults (OA) have unmet healthcare needs. Community-clinic linkage models (CCLM) aim to facilitate healthcare access by linking the community and health sectors. However, research on community-based linkages for community-dwelling OA into the healthcare sector is nascent. CCLM implemented for the general adult population are not necessarily age-friendly. This scoping review aims to characterize programs that are designed to facilitate community-dwelling OA’s access to healthcare through community sector-based linkages. In order to comprehensively survey CCLM designed for community-dwelling OA, the AgeLine (EBSCO) database was searched to review the academic and grey literature regarding programs that link community-dwelling OA from community to clinical sectors. AgeLine focuses on OA, indexing academic and grey literature from disciplines including health sciences, social work, and public policy. 470 publications were included for title/abstract screening; 24 were assessed for full-text eligibility by two independent reviewers. Data were extracted from two publications; exclusions were due to ineligible intervention (n=9) or setting (n=2); insufficient detail (n=6); full text unavailable (n=5). The sole empirical publication (2021) found that pilot implementation of a pilot linkage program between OA and clinicians was significantly associated with improved medication self-management. A final report (1993) on implementation of a supportive service pilot program in New Hampshire public housing found that OA residents and managers benefitted from the program. Results suggest the benefits of age-friendly CCLM for OA but highlight an existing gap in the literature. The results of this review underscore the need for age-friendly CCLM research, practice and innovation.

